# Machine learning prediction models for prognosis of critically ill patients after open-heart surgery

**DOI:** 10.1038/s41598-021-83020-7

**Published:** 2021-02-09

**Authors:** Zhihua Zhong, Xin Yuan, Shizhen Liu, Yuer Yang, Fanna Liu

**Affiliations:** 1 College of Information Science and Technology, Jinan University, Guangzhou, China; 2 College of Traditional Chinese Medicine, Jinan University, Guangzhou, China; 3 Department of Nephrology, The First Affiliated Hospital of Jinan University, 613 W.Huangpu Avenue, Guangzhou, 510632 China; 4 College of Cyber Security, Jinan University, Guangzhou, China

**Keywords:** Disease prevention, Prognosis, Risk factors

## Abstract

We aimed to build up multiple machine learning models to predict 30-days mortality, and 3 complications including septic shock, thrombocytopenia, and liver dysfunction after open-heart surgery. Patients who underwent coronary artery bypass surgery, aortic valve replacement, or other heart-related surgeries between 2001 and 2012 were extracted from MIMIC-III databases. Extreme gradient boosting, random forest, artificial neural network, and logistic regression were employed to build models by utilizing fivefold cross-validation and grid search. Receiver operating characteristic curve, area under curve (AUC), decision curve analysis, test accuracy, F1 score, precision, and recall were applied to access the performance. Among 6844 patients enrolled in this study, 215 patients (3.1%) died within 30 days after surgery, part of patients appeared liver dysfunction (248; 3.6%), septic shock (32; 0.5%), and thrombocytopenia (202; 2.9%). XGBoost, selected to be our final model, achieved the best performance with highest AUC and F1 score. AUC and F1 score of XGBoost for 4 outcomes: 0.88 and 0.58 for 30-days mortality, 0.98 and 0.70 for septic shock, 0.88 and 0.55 for thrombocytopenia, 0.89 and 0.40 for liver dysfunction. We developed a promising model, presented as software, to realize monitoring for patients in ICU and to improve prognosis.

## Introduction

Open-heart surgery is a common surgery in the intensive care unit (ICU) with various complications such as acute kidney injury (AKI), sepsis, septic shock, chronic kidney disease (CKD), pneumonia, thrombocytopenia, and inflammatory responses^1–7^. Lysak et al^8^ showed that because AKI and CKD are prevalent and generate high expenditure, early diagnosis is necessary to prevent these comorbidities from deteriorating. Early predicting comorbidities for critically ill patients after cardiac surgery is vital for patients’ prognosis and doctors’ decision making.

Compared to traditional methods to build a clinical prediction model by using logistic regression (LR), machine learning prediction models have the advantage of higher accuracy and robustness. Traditional algorithms like LR requires researchers to manually select the highly related independent variables X while cutting edge machine learning algorithms can find out the relationship between X and Y automatically. Many researchers tried to construct prediction models for patients who underwent cardiac surgery. Meyer et al^9^ used the recurrent neural network (RNN) to predict mortality, bleeding, and renal failure after patients received heart surgery. Lei et al^10^, Tseng et al^11^, and Lee et al^12^ used acute kidney injury (AKI) as their primary outcome, moreover, Kilic et al^13^ considered prolonged ventilation, reoperation as their prediction objectives. Many researchers paid attention to the most common complications including AKI and sepsis after cardiac surgery, however, the research on other comorbidities such as septic shock, liver dysfunction, severe thrombocytopenia was limited.

Vardon-Bounes et al^14^ suggested that thrombocytopenia, with a prevalence of 50%, is a common hemostatic disorder in ICU, and is associated with bleeding, high illness severity, organ failure, and bad prognosis^15^. Moreover, Kunutsor et al^16^ demonstrated that alanine transaminase (ALT) and aspartate transaminase (AST), as the indicator of liver dysfunction, are inversely associated with coronary heart disease (CHD) and are positively associated with stroke. In addition, Ambrosy et al^17^ showed that the higher the ALT and AST, the lower the survival rate. The increase of transaminase often indicates that the body is in a state of hypoperfusion or hypoxemia. It reminds us that timely intervention is needed, otherwise, patients will have adverse prognoses such as AKI or even death. Font et al^18^ claimed that during septic shock, the body produces a large number of inflammatory cytokines, causing multiple organ failures, such as septic cardiomyopathy, acute respiratory distress syndrome, septic encephalopathy, and other complications. Therefore, early prediction of the occurrence of septic shock is particularly important to reduce the further deterioration of the patient's condition.

Therefore, in this study, we aimed to build up multiple machine learning models to predict several risk factors of prognosis after open-heart surgery. Our primary outcomes were all-cause 30-days mortality, septic shock, severe thrombocytopenia, and liver dysfunction (abnormal AST and ALT).

## Result

### Study population

Among 6844 patients after heart surgery, 5475 (80%) patients were randomly divided into training data and 1369 (20%) patients were in test data. Table 1 showed the characteristics’ differences between these two groups' data, and most of the variables have no significant differences. Among 6844 data enrolled in this study, 219 (3.1%) patients died within 30-days after heart surgery. Septic shock, liver dysfunction, and thrombocytopenia accounts for 32 (0.5%), 248 (3.6%), 202 (2.9%) in respective. Table 2 showed most of the input variables have significant difference between positive samples (ill patients) and negative samples (normal patients) (*P* < 0.05).

**Table 1:** Baseline characteristics and variables 1.

Variables	Total (6844)	Train (5475)	Test (1369)	*P* value	Observed, n (%)
**Demographics**					
Age (years)	66 ± 12	66 ± 12	66 ± 12	0.20	6841(100)
BMI(Mean ± SD)	29 ± 12.5	29 ± 14	29 ± 6	0.02	6544(95.6)
Male, n (%)	4700(69)	3770(69)	930(68)	0.53	6841(100)
Vasopressin, n (%)	337(4.9)	272(4.9)	65(4.7)	0.79	6841(100)
**Lab variables(Mean ± SD)**					
Glucose (mg/dL)	130 ± 19	130 ± 19	130 ± 18	0.48	6766(98.9)
Platelet (K/uL)	191 ± 68	191 ± 68	188 ± 69	0.03	6762(98.8)
Potassium (mEq/L)	4.3 ± 0.3	4.3 ± 0.3	4.3 ± 0.3	0.15	6768(98.9)
Sodium (mEq/L)	137 ± 2.3	137 ± 2.3	137 ± 2.4	0.25	6766(98.9)
WBC (K/uL)	11 ± 3.6	11 ± 3.8	12 ± 2.5	0.06	6761(98.8)
Bicarbonate (mEq/L)	24 ± 2.4	24 ± 2.3	24 ± 2.4	0.12	6622(96.8)
Hematocrit	31 ± 4	31 ± 4	31 ± 4	0.23	6587(96.2)
INR	1.4 ± 0.3	1.4 ± 0.3	1.4 ± 0.3	0.24	6475(94.6)
Lactate (mg/dL)	2.3 ± 1	2.3 ± 1	2.3 ± 1	0.01	4447(64.0)
PCO2 (mmHg)	41 ± 4	41 ± 3.8	41 ± 4	0.38	6687(97.7)
PH	7 ± 0.03	7 ± 0.3	7 ± 0.3	0.33	6700(97.9)
PO2 (mmHg)	236 ± 57	236 ± 57	237 ± 57	0.34	6687(97.7)
SPO2 (%)	98 ± 1.5	98 ± 1.5	98 ± 1.3	0.48	6716(98.1)
Base excess (mEq/L)	2.3 ± 1	2.3 ± 1	2.3 ± 1	0.15	5657(82.7)
Total CO2 (mEq/L)	25 ± 2	25 ± 2	25 ± 2	0.31	6687(97.7)
Calcium (mg/dL)	1 ± 0.2	1 ± 0.2	1 ± 0.1	0.42	6557(95.8)
Creatinine (mg/dL)	1.1 ± 0.8	1.1 ± 0.8	1.1 ± 0.7	0.32	6763(98.8)
Urea nitrogen (mg/dL)	22 ± 12	22 ± 12	22 ± 12	0.19	6763(98.8)
Anion gap (mEq/L)	12 ± 2.5	12 ± 2.5	12 ± 2.5	0.14	6013(87.9)
Hemoglobin (g/dL)	10 ± 1.5	10 ± 1.4	10 ± 1.4	0.15	6763(98.8)
PTT (s)	41 ± 16	41 ± 16	41 ± 15	0.25	6491(94.9)
PT (s)	15 ± 2.7	15 ± 2.6	15 ± 3.1	0.47	6477(94.7)
**Vital signs(Mean ± SD)**					
Heart rate (bpm)	84 ± 10	84 ± 10	84 ± 10	0.34	6718(98.2)
Systolic BP (mmHg)	112 ± 9	112 ± 9	112 ± 9	0.05	6709(98.1)
Diastolic BP (mmHg)	56 ± 6	56 ± 6	57 ± 6	0.13	6709(98.1)
Respiratory rate (bpm)	17 ± 3	17 ± 3	17 ± 3	0.24	6716(98.2)
Body temperature (°C)	37 ± 0.5	37 ± 0.5	37 ± 0.5	0.26	6197(90.6)
**Comorbidities, n(%)**					
CHD	5029(73)	4003(73)	1026(75)	0.18	6841(100)
Diabetes	2160(32)	1738(32)	422(31)	0.57	6841(100)
History of stroke	4(0.06)	4(0.07)	0(0)	0.70	6841(100)
Urine output(Mean ± SD)	2262 ± 1123	2265 ± 1137	2249 ± 1062	0.48	6688(97.8)
**Primary outcomes, n(%)**					
30-days mortality	215(3.1)	170(3.1)	45(3.3)	0.80	6841(100)
Liver dysfunction	248(3.6)	197(3.6)	51(3.7)	0.99	6841(100)
Septic shock	32(0.5)	25(0.5)	7(0.5)	0.96	6841(100)
Thrombocytopenia	202(2.9)	153(2.8)	49(3.5)	0.99	6841(100)

Abbreviations: *BMI* body mass index, *CHD* coronary heart disease, *INR* international normalized ratio, *PCO2* partial pressure of carbon dioxide, *PO2* partial pressure of oxygen, *PTT* partial thromboplastin time, *PT* prothrombin time, *SPO2* oxygen saturation, *WBC* white blood cell. All laboratory variables and vital signs mentioned above were measured by the mean value during hospitalization.

Chi-square test and Wilcoxon rank-sum test were used to compare the differences of categorical and continuous variables respectively.

**Table 2:** Baseline characteristics and variables 2.

Variables	*P* value of mortality	*P* value of thrombocytopenia	*P* value of septic shock	*P* value of liver dysfunction
**Demographics**				
Age	< 0.001	< 0.001	0.007	0.032
Body mass index	0.459	0.025	0.005	0.453
Male	0.226	0.067	0.953	0.536
Vasopressin	< 0.001	< 0.001	< 0.001	< 0.001
**Lab variables**				
Glucose	< 0.001	0.008	0.003	0.065
Platelet	< 0.001	< 0.001	0.057	< 0.001
Potassium	0.187	< 0.001	0.255	< 0.001
Sodium	0.001	0.064	0.251	0.007
White blood cell	< 0.001	0.011	< 0.001	< 0.001
Bicarbonate	< 0.001	< 0.001	< 0.001	< 0.001
Hematocrit	< 0.001	< 0.001	0.293	0.092
International normalized ratio	< 0.001	0.005	0.203	0.002
Lactate	< 0.001	< 0.001	0.017	< 0.001
Partial pressure of carbon dioxide	0.37	< 0.001	0.346	0.393
PH	< 0.001	0.001	< 0.001	< 0.001
Partial pressure of oxygen	< 0.001	0.001	< 0.001	< 0.001
Oxygen saturation	< 0.001	0.003	0.015	< 0.001
Base excess	< 0.001	0.106	0.143	< 0.001
Total CO2	< 0.001	< 0.001	< 0.001	< 0.001
Calcium	< 0.001	0.29	< 0.001	< 0.001
Creatinine	< 0.001	< 0.001	< 0.001	< 0.001
Urea nitrogen	< 0.001	< 0.001	< 0.001	< 0.001
Anion gap	< 0.001	0.442	< 0.001	< 0.001
Hemoglobin	< 0.001	< 0.001	0.374	0.142
Partial thromboplastin time	< 0.001	< 0.001	0.437	< 0.001
Prothrombin time	< 0.001	< 0.001	0.344	0.003
**Vital signs**				
Heart rate	< 0.001	0.097	0.123	0.126
Systolic BP	< 0.001	0.012	< 0.001	< 0.001
Diastolic BP	0.007	0.014	0.007	0.346
Respiratory rate	< 0.001	< 0.001	< 0.001	< 0.001
Body temperature	< 0.001	0.071	0.008	0.129
**Comorbidities**				
Coronary heart disease	0.051	< 0.001	0.073	0.658
Diabetes	0.423	0.901	0.231	0.092
History of stroke	0.283	0.532	< 0.001	0.343
Urine output	< 0.001	< 0.001	< 0.001	< 0.001

*P* value of outcomes presents whether a variable has significant difference between positive samples (ill patients) and negative samples (normal patients).

### Machine learning models’ performance

**Figure 1:**
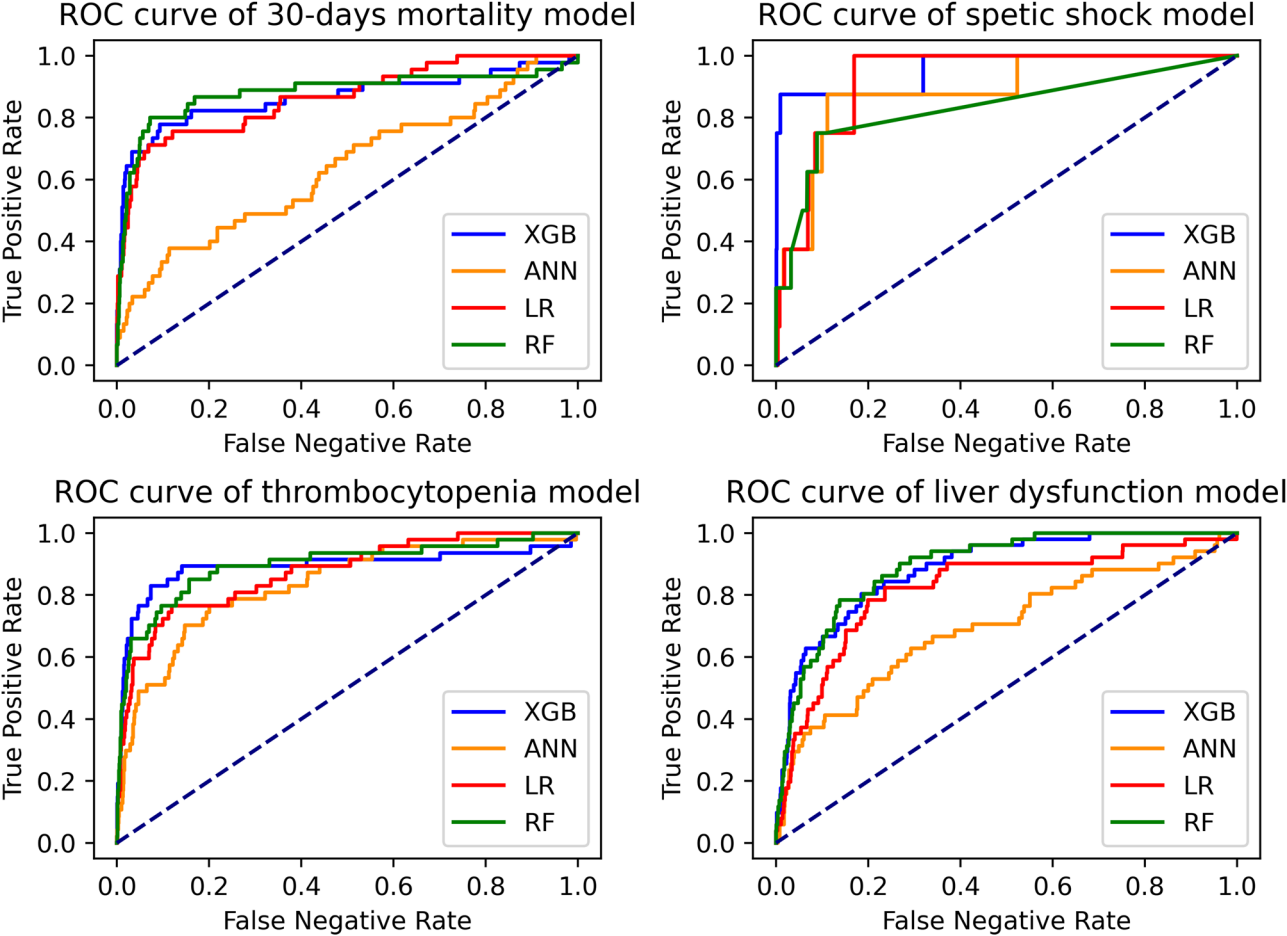
ROC curve of 4 outcomes.

Accuracy, area under the curve (AUC), F1 score, precision, and recall of four models of all complications were shown in Table 3, and ROC curves of 4 primary outcomes were plotted in Fig. 1.

**Table 3:** Model evaluation.

Outcomes	Model	Accuracy	AUC	F1 score	Precision	Recall
30-days mortalit	XGBoost	0.97	0.90	0.58	0.58	0.58
	RF	0.97	0.88	0.44	0.50	0.40
	LR	0.81	0.86	0.21	0.12	0.75
	ANN	0.97	0.64	0.12	0.75	0.07
Septic shock	XGBoost	0.99	0.96	0.70	0.67	0.75
	RF	0.99	0.81	0.25	1.00	0.14
	LR	0.79	0.93	0.05	0.02	1.00
	ANN	0.99	0.88	0.13	0.13	0.14
Thrombocytopenia	XGBoost	0.96	0.89	0.55	0.45	0.72
	RF	0.97	0.89	0.37	0.56	0.27
	LR	0.83	0.87	0.23	0.14	0.76
	ANN	0.89	0.83	0.24	0.16	0.51
Liver dysfunction	XGBoost	0.94	0.89	0.40	0.32	0.53
	RF	0.96	0.89	0.20	0.39	0.14
	LR	0.81	0.82	0.22	0.13	0.70
	ANN	0.96	0.70	0	0	0

Abbreviations: *XGBoost* extreme gradient boosting, *RF* random forest, *LR* logistic regression, *ANN* artificial neural network, *AUC* area under curve.

XGBoost (AUC: 0.99; F1 score 0.70 for septic shock; AUC: 0.88; F1 score 0.58 for 30-days mortality; AUC: 0.88; F1 score 0.55 for thrombocytopenia; AUC: 0.89; F1 score 0.40 for liver dysfunction) achieved the highest AUC and F1 score, which means it is the most robust model.

**Figure 2:**
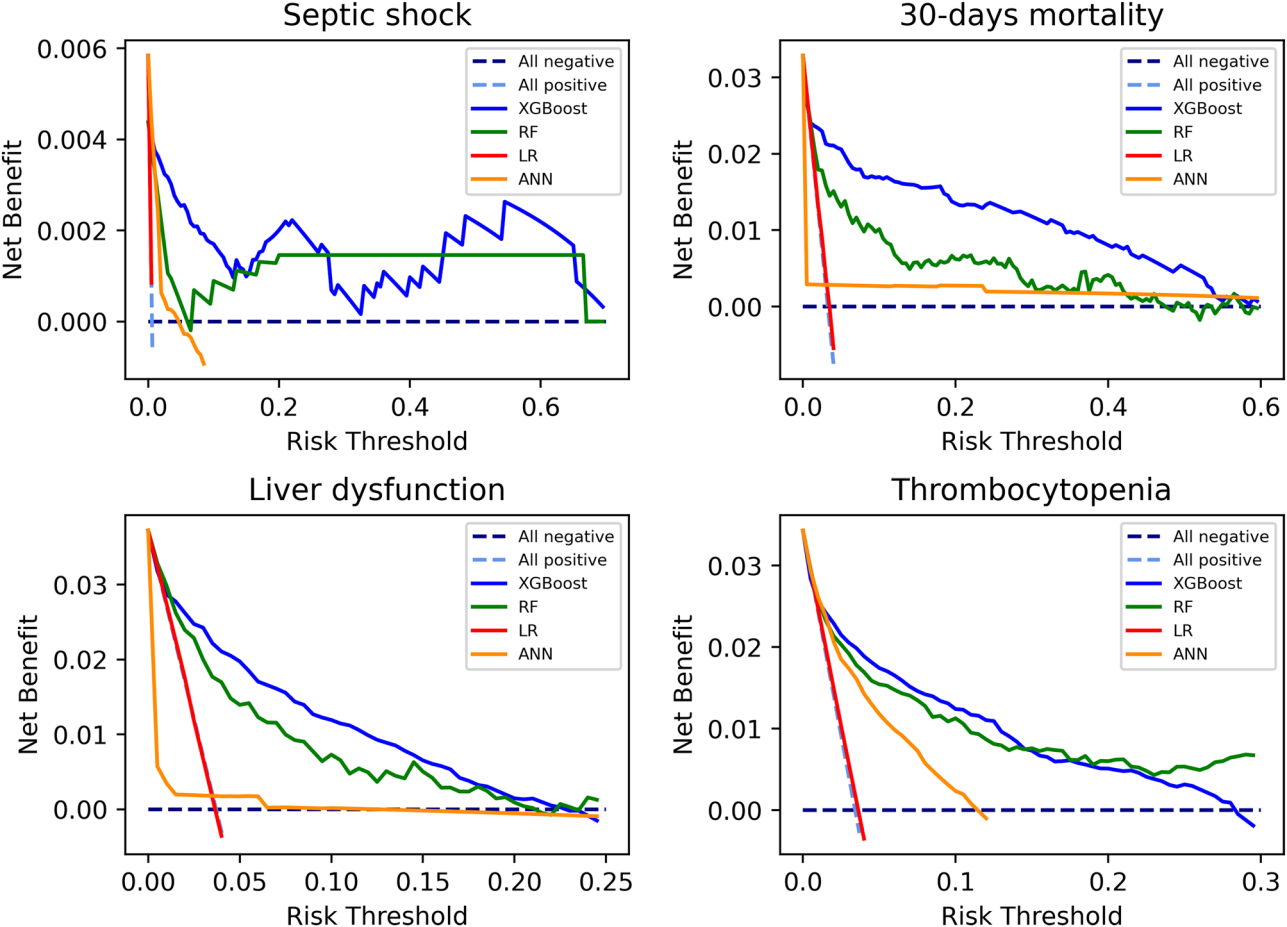
Decision curve analysis of 4 outcomes.

**Figure 3:**
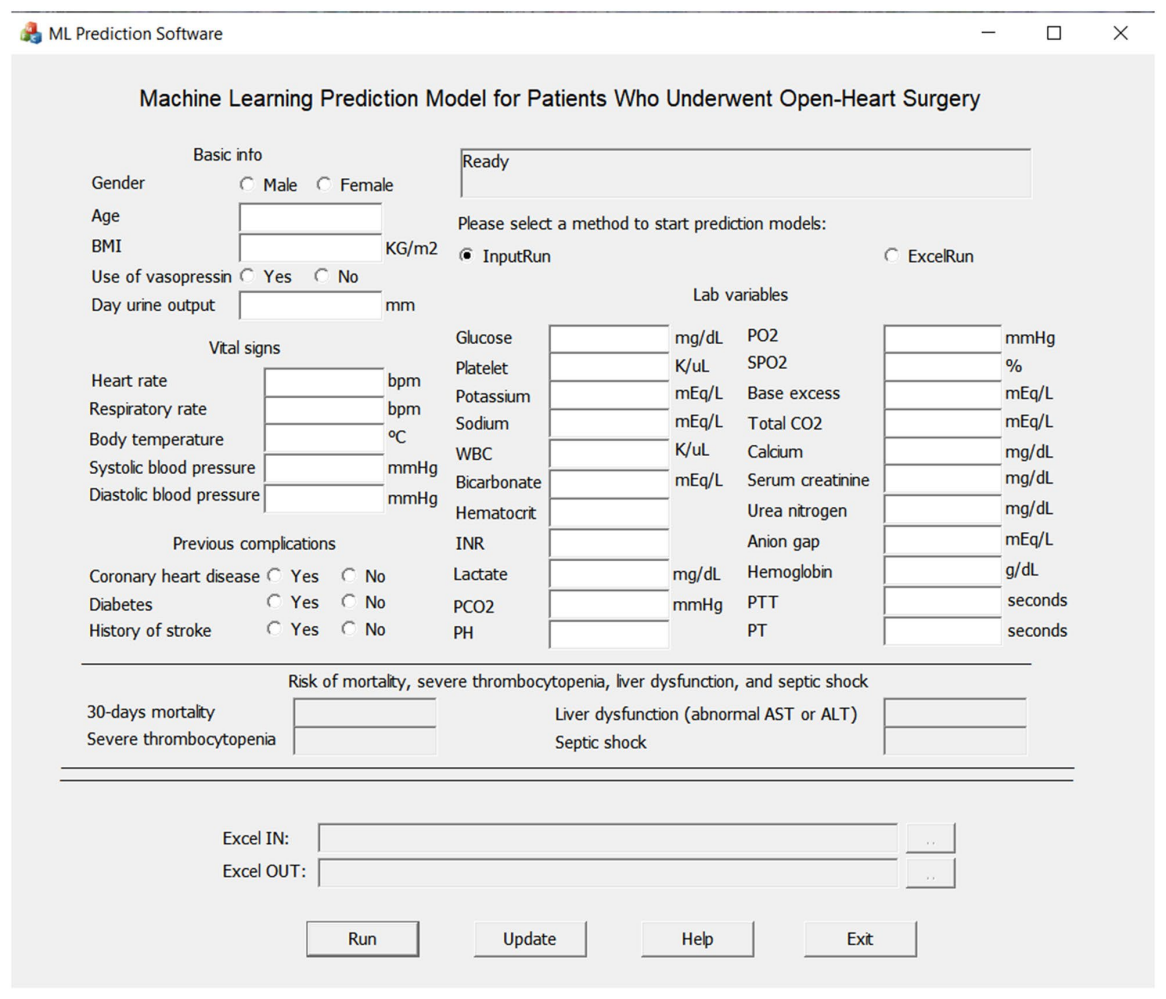
Windows 10 software for patients who underwent open-heart surgery.

Compared to other algorithms, XGBoost has overall better performance in terms of AUC, test accuracy, and F1 score in respective. In Fig. 2, decision curve analysis showed that, in terms of net benefit, XGBoost and RF were better than LR and ANN. Besides, XGBoost is slightly better than RF. Therefore, we selected XGBoost model as our final model in this study, and based on XGBoost model files we built up a Windows 10 software, which can find the download link from the website https://github.com/Zhihua-PredictionModel/ML-Prediction-Model, to present our research results as shown in Fig. 3. Source files of XGBoost for 4 outcomes from Sklearn were also uploaded to the website. Other researchers or programmers can easily apply these trained model files (“.model” file can be loaded by joblib, a package in Python) to the practical customized use of prediction.

The top 5 predictors that influenced the decision making of XGBoost were calculated as shown in Table 4. The first, second, and third predictors of 4 outcomes are as follow. 30-days mortality: vasopressin (first), PH (second), and creatinine (third); septic shock: hemoglobin, hematocrit, and lactate; severe thrombocytopenia: vasopressin, bicarbonate, lactate; liver dysfunction: partial thromboplastin time, gender, partial pressure of oxygen.

**Table 4:** Feature importance outputted by XGBoost.

Outcomes	First	Second	Third	Fourth	Fifth
30-days mortality	Vasopressin (0.0940)	PH (0.0553)	Creatinine (0.0504)	Lactate (0.0415)	Platelet (0.0414)
Septic shock	Hemoglobin (0.1590)	Hematocrit (0.1400)	Lactate (0.0818)	SPO2 (0.0737)	Respiratory rate (0.0713)
Thrombocytopenia	Vasopressin (0.1486)	Bicarbonate (0.0767)	Lactate (0.0547)	Platelet (0.0452)	Systolic BP (0.0405)
Liver dysfunction	PTT (0.1116)	Gender (0.0659)	PO2 (0.0539)	Platelet (0.0481)	Creatinine (0.0452)

Abbreviations: *PH* potential of hydrogen, *SPO2* oxygen saturation, *Systolic BP* systolic blood pressure, *PTT* partial thromboplastin time, *PO2* partial pressure of oxygen.

Values in parentheses were frequency score of each independent variable, which represents the ratio between the number of times a variable appears in the leaf node and the number of times all variables appear in the leaf node.

## Discussion

In our study, four machine learning models were constructed and compared for 30-days mortality and 3 comorbidities after heart-related surgery. Other researchers also conducted many researches on the prediction model for patients. Based on 2010 patients in the database of Seoul National University Hospital, Lee et al^12^ found that among machine learning algorithms including decision tree, support vector machine, and random forest, XGBoost (Test accuracy: 0.74; AUC: 0.78) has the best performance to predict AKI after cardiac surgery and a website was created to process patients’ data in real-time. Kilic et al^13^ also applied XGBoost to predict multiple complications, including operative mortality (AUC: 0.771), renal failure (AUC: 0.776), prolonged ventilation (AUC: 0.739), reoperation (AUC: 0.637), stroke (AUC: 0.684), and deep sternal wound infection (AUC: 0.599), for adult patients after surgical aortic valve replacement in the Society of Thoracic Surgeons National Database. In addition, other researchers usually paid attention to common complications such as AKI, sepsis, and hospital mortality. However, researches on other complications were limited. Therefore, our study managed to predict 30-days mortality, septic shock, liver dysfunction, and severe thrombocytopenia which is also important for patients’ prognosis.

Several predictors of different comorbidities were outputted by XGBoost. According to Table 4, among 4 primary outcomes, lactate and platelet appeared 3 times, vasopressin, creatinine, platelet, appeared 2 times, which means they were the important factors for our outcomes. Models built by Kilic et al^13^ also showed that creatinine is an important factor to predict mortality, renal failure after heart surgery.

Our study has some limitations. Firstly, all experiments were conducted on a clinical database of critically ill patients called MIMIC-III, which means our machine learning models may have a good performance on those who are critically ill and are living in America. However, models may not work that well on people living in other regions. Therefore, further study is needed to obtain as much as possible data from various databases to construct a more comprehensive model that can work well on any population in any area.

Secondly, sample imbalance problem occurred in the experiment. There is a trade-off between accuracy, F1 score, precision, recall, and AUC because medical data usually are highly unbalanced that among 100 patients, there may be three positive samples and 97 negative samples (normal samples). In this situation, ML algorithms tend to classify samples into the class with most data. Therefore, we adjusted the weight of the positive samples of complications in the loss function and it improved precision, recall, and F1 score of models at the cost of reducing AUC and accuracy. And by setting other hyperparameters and using subsample, we make XGBoost keep a good balance between precision and recall. Facing with unbalanced medical data, how to improve accuracy, F1 score, and AUC simultaneously as much as possible remains an open problem.

In conclusion, four machine learning algorithms were built to predict 30-days mortality and 3 comorbidities after open-heart surgery. XGBoost model was the most robust model with the highest AUC, F1 score, and net benefit. Besides, Windows 10 software was created and is available on the website mentioned above for clinical staff. Moreover, multiple predictors outputted by XGBoost model indicated the relevance between these factors and comorbidities, and generated a hypothesis. Besides, whether these factors can be independent biochemical indexes remain open problems.

## Methods

### Data source and participants

Medical Information Mart for Intensive Care (MIMIC-III) is a freely available database containing critically ill patients who were admitted to the ICU of the Beth Israel Deaconess Medical Center between 2001 and 2012^19^. Those who were under coronary artery bypass surgery, aortic valve replacement, or insertion of the implantable heart assist system (including ICD9 code 3961, 3615, 3612, 8872, 3521, 6311, 3522, 3614, 3733, 3524) were enrolled in this study. 6844 related samples were extracted from MIMIC-III clinical database by using PostgreSQL and Python 3 (version 3.7.8).

### Definition and primary outcomes

The primary outcomes were 30-days mortality, and three comorbidities including septic shock, liver dysfunction, and severe thrombocytopenia after heart-related surgery. 30-days mortality was defined as death after discharge from ICU within 30 days. A patient will be marked as liver dysfunction if his/her first test value of aspartate transaminase (AST) and alanine transaminase (ALT) was normal (10–45 IU/L for ALT; 10–35 IU/L for AST) and values, tested later, of AST or ALT were greater than the max normal value (45 IU/L for ALT; 37 for AST)^20^. Severe thrombocytopenia was considered as that first platelet count was higher than 50 K/uL and one of later platelet count was lesser than 50 K/uL^14^. Considering septic shock is a severe disease with acute symptoms, it was diagnosed by ICD-9 code (785.52) in MIMIC-III database^21^. Only data of the first time ICU admission for each patient was considered.

### Machine learning models

Logistic regression (LR) is a classic classification algorithm that makes a linear combination of input variables and uses the sigmoid function to output a probability. Main LR hyperparameter is C.

Neurons in artificial neural network (ANN) make a linear combination of the output value from the upper layers’ neurons, pass it through sigmoid functions, and finally output a value to the next neurons^22^. The width and depth of hidden layers influence the performance of ANN.

Compared to a single classifier, the ensemble learning algorithm random forest (RF), merging multiple weak classifiers to a strong classifier, showed a more powerful performance in the classification task^23^. Main hyperparameters are n_estimators, max_depth, and max_leaf_nodes.

Extreme gradient boosting (XGBoost) is also an ensemble model of decision trees^24^. Main parameters are n_estimators, max_depth, reg_lambda, gamma, min_child_weight, scale_pos_weight (when samples are unbalanced, this parameter can change the weight of positive samples in loss function), max_delta_step, and subsample.

### Statistical method

35 input variables including demographics, use of vasopressin, laboratory variables, vital signs, comorbidities, urine output of the first day, and 4 output variables were extracted from the database as shown in Table 1. Some important variables such as fraction of inspiration O2 (Fio2) were excluded due to too much missing data. Variables that have less than 40% missing data were retained^25^. All missing values were filled with the average value of this variable. A statistical method called winsorization was used to deal with the outliers.

**Figure 4:**
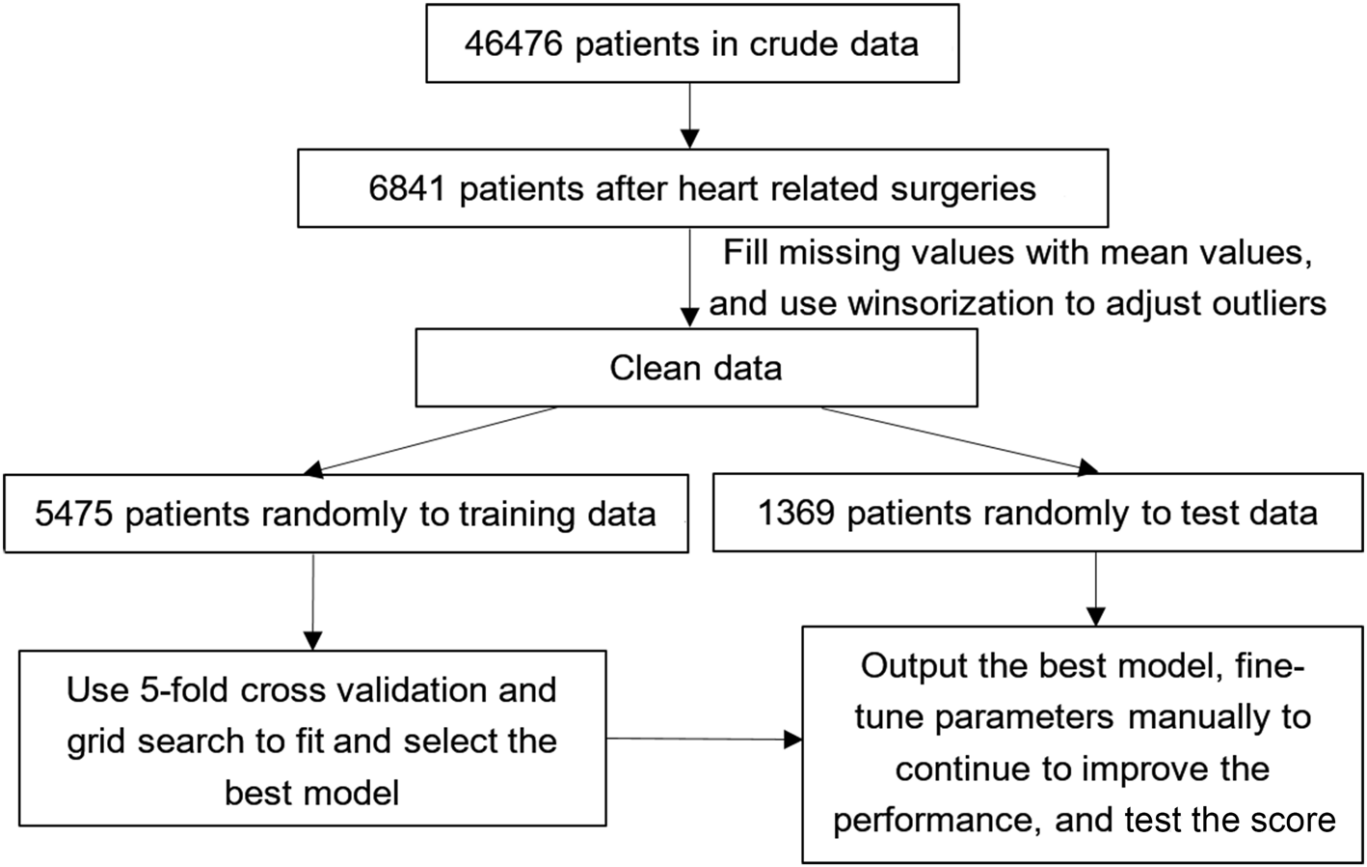
Flow chart of data process.

Figure 4 showed the flow chart of data process. After that, 6844 samples were randomly divided into training data (5475), and test data (1369) in a ratio of 80–20%. Chi-square test and Wilcoxon rank-sum test were used to compare the differences of categorical and continuous variables respectively. They were employed to compare the differences between training data and test data to make sure the distributions of two datasets were as same as possible. In Table 1, *P* value was calculated and *P* > 0.05 was considered there were no significant distribution differences between training data and test data. In Table 2, Chi-square test and Wilcoxon rank-sum test were used to compare the difference between positive samples and negative samples to observe the correlation between the independent variables X and outcome variables Y. *P* < 0.05 was considered there was strong correlation.

### Machine learning models training

Training data were evenly split into 5 parts that 4 parts were used to train a model of a certain hyperparameter, and the remaining one, also called validation set, was used to test the performance of this parameter. This process will conduct 5 times to gain 5 validation scores and the average score was used to evaluate the performance of the model. Data scientists usually call this method fivefold cross-validation which usually was used to select the best hyperparameter. 4 machine learning algorithms including LR, ANN, RF, and XGBoost were employed to fit the data, and all of these models have many hyperparameters that need to be specified as shown in Table 5. By applying grid search techniques, various kinds of parameters were searched automatically in Python and the best one was selected. Each model has its own best parameter. By comparing four models' performance, the best one, XGBoost, was picked up to be the final model of our study. We continued to fine-turn hyperparameters of XGBoost manually to obtain a better performance.

**Table 5:** Candidate parameters for grid search and fine-tune of parameters.

Algorithms	Candidate parameters
Logistic regression	C: [0.01, 0.1, 1, 10] class_weight: [‘balanced’]
Artificial neural network	hidden_layer_sizes: [(50, 50), (100, 100), (150, 150)]
Random forest	n_estimators: [30, 60 ,90] max_depth: [30, 60, 90] max_leaf_nodes: [30, 60 ,90] class_weight: [‘balanced’]
XGBoost	n_estimators: [3,30,60] max_depth: [3, 30] reg_lambda: [0.1, 10] gamma: [0.1, 10] min_child_weight: [0.2, 8, 20] scale_pos_weight: [3,30,300,3000,3300,3600,3900,4000,5000,6000,7000,8000] subsample:[0.3, 0.6, 1] max_delta_step:[0, 3]

These parameters came from Python machine learning package called Sklearn.

It will overestimate the model performance if just using the validation set and its score to evaluate the model, and because of that, test data, divided at the beginning, will be utilized to obtain a final score whose results were presented in Table 3. Besides, decision curve analysis was also applied to evaluate the model as shown in Fig. 2. All machine learning experiments were conducted on Python (version 3.7.8).

## Data Availability

Original data were extracted from the MIMIC-III database by Z.Z., the first author, who passed the online training and obtained access to the database, https://mimic.mit.edu. If needed, related data of this article can be obtained by contacting F.L., the corresponding author, on reasonable request.
